# Quality and Safety Management of Advanced Medical Technologies in Homecare in The Netherlands: A Qualitative Study on Consensus Development Regarding Approaches and Continuing Professional Education

**DOI:** 10.3390/healthcare14040529

**Published:** 2026-02-20

**Authors:** Ingrid ten Haken, Somaya Ben Allouch, Wim H. van Harten

**Affiliations:** 1 Research Group Technology, Health and Care, Saxion University of Applied Sciences, 7513 AB Enschede, The Netherlands; 2 Research Group Digital Life, Amsterdam University of Applied Sciences, 1091 GH Amsterdam, The Netherlands; 3 Informatics Institute, University of Amsterdam, 1098 XH Amsterdam, The Netherlands; 4 Department Health Technology and Service Research, Faculty of Behavioural, Management and Social Sciences, University of Twente, 7522 NB Enschede, The Netherlands

**Keywords:** medical technology, homecare, nursing education, patient safety, qualitative study, risk management

## Abstract

**Background/Objectives**: Dutch legislation sets requirements for the safe reporting of and learning from incidents. It also specifies the required competence of nurses in using medical technology. However, not all certified homecare nurses are adequately trained in patient safety. Patient safety management is reflected at different levels within homecare organisations. This study aimed to report on initial consensus among homecare nurses on responsibilities in quality and safety management at organisational, team and individual levels. It also explored nurses’ educational needs related to the use of advanced medical technologies (AMTs) in homecare. **Methods**: An exploratory qualitative study using consensus-oriented member checking was conducted. Building on research into incidents and safety management practices of AMTs, two semi-structured group interviews were conducted online with 11 homecare nurses from across the Netherlands. In a second round, feedback and comments were solicited on the resulting conclusions and statements in writing. **Results**: Distinguishing between high-risk and low-risk incident reports enhances the efficiency and effectiveness of safety management for AMTs in homecare. Team-based discussions increase the likelihood of incident reporting. Nurses advocate for periodic, mandatory assessments for technical homecare teams, conducted by an external body. They also emphasise individual responsibility for maintaining up-to-date knowledge and skills and taking action accordingly. **Conclusions**: In this study, key statements on which Dutch technical homecare nurses reached consensus are presented. The results underscore the importance of a safe organisational and team culture for incident reporting, as well as the need for an effective and efficient incident management system at a team level. An effective learning organisation contributes to enhancing patient safety.

## 1. Introduction

With the growth of advanced medical technologies (AMTs) in homecare, such as infusion therapy or morphine pumps, there has also been an increased focus on quality and patient safety in its use [1,2,3]. In recent years, we have conducted extensive research on this topic. Key findings are that there is a lack of research exploring user experiences and on education of nurses in AMTs, on risk management and patient safety [4]. There is considerable formal under-reporting of incidents involving AMTs in homecare [5]. We also found aspects relating to education that influence patient safety risks, such as skills not always being tested and not all nurses formally registering their competencies [6,7]. An overarching conclusion is that nurses generally possess a strong awareness of quality and patient safety; however, formal systems do not always align with their working methods.

Dutch legislation requires organisations to develop contingent quality systems and frameworks for the safe reporting of and learning from incidents [8]. Regarding the use of medical technology, clear definition of tasks, authorities, responsibilities and competency requirements are required [9]. One way for nurses to demonstrate their competence and proficiency in medical technology is through certification [10]. However, not all certified nurses are adequately trained in patient safety. Therefore, it is advocated that education in patient safety be aligned across different levels of healthcare [6,11].

Patient safety is a responsibility that operates at various levels within homecare organisations [12,13]. To enhance patient safety, the establishment of an effective learning and resilient organisation is crucial [14,15,16]. The primary approach is based on incident reporting, with a retrospective focus on what goes wrong and identifying underlying causes (Safety I) [17,18]. However, there is also a shift towards a prospective focus on what goes well and improving the organisation’s learning processes [19,20,21,22]. The Safety II approach facilitates a deeper understanding of nurses’ ability to act flexibly and creatively in complex situations encountered in daily practice, thereby preventing incidents and enhancing patient safety. It has the potential to transform healthcare organisations into more effective learning environments [15,23,24,25,26]. However, some practical workarounds are labour-intensive and not scalable; more scientific evidence is needed to support this approach [23,27].

Aligning different organisational levels in quality and safety management systems in homecare is a challenge. Formal organisational policy and team and individual functioning was found to differ in many instances and the growing use of AMTs may complicate this further. To our knowledge, there is a lack of clear consistency in this field, both nationally and internationally. Using the findings of our earlier research, the objective of this study was to report on initial consensus among homecare nurses on organisational, team and individual responsibilities in quality and safety management, as well as the educational needs related to the use of AMTs in homecare.

## 2. Materials and Methods

### 2.1. Study Design, Setting and Participants

An exploratory qualitative study using consensus-oriented member checking was conducted [28,29]. The participants were homecare nurses, who work with AMTs, from a sample of homecare organisations from across the Netherlands. Two semi-structured group interviews were used. The aim of this was to achieve consensus among participants regarding key findings.

Participants were sampled from homecare nurses who had participated in previous studies in the same research project [5,6]. This group was representative of the Netherlands in several ways. This included specialised homecare nurses (who perform specific care tasks for patients at home using AMTs), general homecare nurses (who usually perform various care tasks for patients at home, including care with AMTs if necessary), distribution across the country and also with regard to gender. Nurses were eligible to participate if they were working in practice with several AMTs, including the most frequently used technologies, such as infusion therapy, parenteral nutrition and/or morphine pumps.

The aim was to include 8–12 homecare nurses per group [29]. Of 238 approached, 57 homecare nurses responded to the invitation and reminder email and 25 were willing to participate. Five were unavailable for neither one of the sessions on the two specified dates, including two generalist homecare nurses. Each group interview was assigned 10 respondents. However, at the last moment, some respondents withdrew or failed to show; a few participants indicated that this was for personal reasons, but the reasons for the others are unknown. Ultimately, seven and four participants participated in two online group interviews in February 2024. All participants had extensive experience with AMTs, and the organisations they represented constitute a substantial share of Dutch homecare providers in this field, with representation from all geographic regions of the Netherlands.

### 2.2. Data Collection

A semi-structured discussion guideline was used for both group interviews [30,31], structured around the four topics ‘safety management systems’, ‘incident reporting at team level’, ‘safety II in technical homecare teams’, and ‘ensuring the competence and authority of homecare nurses’ (see Supplementary File SA). This was pilot tested. Some questions were reformulated or better explained with a short introduction. An audio-visual recording was made of each group session, which lasted approximately 90 min and was led by the principal researcher (#1). The participants were informed about the study and their rights prior to the group meetings by means of an information letter. Participants gave written informed consent in advance. Data collection and analysis were conducted iteratively. The second group interview yielded little additional information and no new themes emerged compared with the first, indicating that data saturation had been reached [32].

### 2.3. Data Analysis

The recordings were transcribed verbatim. In the first phase, a thematic analysis [33] was used to organise, code and sort the data. The transcripts of both group interviews were thoroughly reviewed by two authors (#1 and 3) to familiarise themselves with the data. Both a deductive and inductive approach were used, using the four themes in the topic guide as a starting point. Initial codes were defined by the two authors and discussed until consensus was reached. Sub-themes that emerged during the analysis were subsequently coded and refined, using Atlas.ti software 22–23.3.4. Codes were grouped into overarching themes. The key findings were subsequently derived from the core content of the themes, with preliminary statements formulated reflecting this central content.

In the second phase, the preliminary statements on the topics were presented to all 25 homecare nurses who had initially been willing to participate in the group interviews. Our objective was to achieve maximal consensus for each statement by revising formulations per step. For the purpose of this study, consensus was defined as qualitative agreement among those who responded to each statement. Consensus was not further quantified, as the focus was on the interpretation of the themes. Following a reminder, 17 did not respond, 5 agreed without providing additional comments, and 3 nurses provided substantive feedback. The responses mainly concerned medication safety in relation to safety management systems, the presence of an effective incident reporting system, and the discussion of incident reports at a team level. In addition, a new model of training within the organisation was mentioned, as well as the need for policies that are uniform regarding nursing procedures. Where revisions to the wording of a statement were proposed, relevant comments were incorporated into the statements where applicable. In the subsequent round, consensus was reached on the revised versions, all statements were validated and none were discarded. Final statements were formulated as the main conclusions to the themes.

### 2.4. Ethics

According to Dutch legislation, no ethical approval was needed in this case, as this study was not part of the Medical Research Involving Human Subjects Act, https://english.ccmo.nl/investigators/legal-framework-for-medical-scientific-research/your-research-is-it-subject-to-the-wmo-or-not (accessed on 22 April 2024). The study was carried out in accordance with the ethical guidelines of the University of Twente and the Saxion University of Applied Sciences. Participants were informed of the purpose of the study, how the data were handled and their rights prior to the interviews. Written informed consent was obtained from all participants.

## 3. Results

### 3.1. Participant Characteristics

The participants in the group interviews each had a minimum of five years’ practical experience with AMTs. All participants were female and were working in a specialised nursing team that provides homecare involving AMTs. Some nurses had side activities, such as participating in the development of nursing courses or guidelines, administering tests or providing training (see Supplementary File SB).

### 3.2. Safety Management System Regarding Advanced Medical Technologies

The great majority of nurses assumes that there is a safety management system at the organisational level, but they do not directly utilise it in practice and do not miss it.

*It is, yes, although I don’t know whether there’s really a need for it, because I don’t miss it at the moment. Let me just… Perhaps that’s a bit simplistic, but I don’t have the feeling that there are any processes that haven’t been described which would prevent me from working safely.*

(P3)

*I also think that a great deal is arranged for us at a high level, through a safety management system. I wouldn’t know the details, but organisation-wide they will have that very well in place.*

(P8)

However, nurses consider training and testing as essential components of a safety management system concerning AMTs. Nurses find the national Vilans protocols (Vilans is the national knowledge organisation in the Netherlands for care and support; Vilans Protocols are an online environment for nurses and caregivers that allows users to directly access the correct work instructions from any device when needed.) [34], framework agreements with other healthcare institutions/hospitals, and the professional code more directive for safe practices with AMTs than a safety management system. This concerns not only the safety of the medical technology itself but also the preconditions under which it is used.

*Yes, I believe this is also because I always take into account whether the patient is safe. When I perform a procedure, it reflects a degree of professionalism. This is also because I work according to protocols, which, in my view, incorporate all these considerations.*

(P3)

In the context of safety management regarding AMTs, nurses consider an adequate incident reporting system with procedures essential. Reports can take the form of a MIM (Employee Incident Report), a MIC (Client Incident Report), or a TIM (Transmural Incident Report). The procedures are general in nature and not specifically directed at AMTs.

### 3.3. Levels and Processes Regarding Incident Reporting

According to nurses, high-risk incidents—with more serious consequences for patients—must always be reported and handled according to the organisation’s formal protocol. An example of a high-risk incident is when the incident results in a hospital admission. Views differ, however, when it comes to low-risk incidents, for example, incidents in which there is no harm to the patient. Some nurses believe these should be discussed only within the team, as this increases the likelihood of reporting due to a greater sense of safety at the team level.

*I think that would increase the likelihood of more being reported, because at team level people often feel safer passing things on. If you were to make a distinction, that would of course be difficult: what is a high-risk report and what is a low-risk one? That differs for everyone, but I do think the number of reports would increase.*

(P6)

Others argue that even low-risk incidents should be formally reported, as comprehensive reporting provides more data, allowing for identification and measurements of all incidents, more effective action planning, and maintaining overall alertness.

*By reporting everything, you can gain a clearer understanding of what is happening and implement actions more effectively. Naturally, some incidents are less severe or have no direct consequences, but it is still important to document them all. If you do not have a comprehensive overview, you risk overlooking certain aspects, potentially leading to errors.*

(P7)

This approach also provides a complete overview of all teams, ensuring organisational transparency.

*As an organisation, transparency is essential…. Teams that report very few incidents are not necessarily performing exceptionally well; rather, it indicates a lack of alertness. Incidents occur everywhere, after all.*

(P4)

Formal reporting and discussing incidents within the team are regarded as complementary. Advantages of discussing incidents within the team include direct interaction, immediate discussion or feedback, gaining tips for improvement, and experiencing support. However, openness and a sense of safety within the team are seen as crucial. While discussing incidents within the team is considered very important, nurses express a desire for more time to delve deeper into situations and learn from them.

*As a team, we see it as an important value not to remain solely at the practical level, but to discuss the underlying layer together as well. That can be facilitated, for example, by planning, allowing or enabling regular moral deliberation. Unfortunately, in my organisation that is lacking. Yes.*

(P3)

### 3.4. Safety II in Technical Homecare Teams

Within teams, incidents are discussed more frequently than successes, with a greater focus on patient satisfaction. When positive examples do arise, this tends to be implicit and not consciously reflected upon by the team.

*Yes, but you know, you notice, we’re of course all so trained in providing professional care that it’s already so normal for us, so you don’t mention all of it. You very quickly tend to focus on the improvement actions rather than the positive aspects.*

(P4)

Nurses do see the value in systematically discussing positive examples, but this requires dedicated meeting time.

Some teams take a moment each day where members ask how everyone is doing and can share both positive and negative experiences with colleagues. During these sessions, successes are highlighted, including instances where team members feel proud or satisfied with how they handled complex situations. These daily discussions help maintain vigilance and continuous improvement, in nurses’ views.

*I also want to learn indirectly, through others. I believe you’re kept on your toes by everything and everyone—hospitals, policy staff, and quality officers.*

(P8)

Another participating nurse added:

*How are you kept on your toes? Well, actually, by discussing experiences on a daily basis.*

(P10)

Nurses view adaptability as a core skill necessary for working in a Technical Homecare Team, given the constant variations in their work. Flexibility is also emphasised in their job descriptions, requiring them to work independently, devise solutions and exhibit a degree of creativity.

*Well, I think that anyone working in home care, whether in regular homecare or a technical team, needs to have certain personality traits to be able to operate there at all. In the past, I’ve often seen that many people struggle in homecare because, well, they simply can’t manage it—the independent working and coming up with solutions themselves. And yes, being on your own like that isn’t something everyone can handle.*

(P6)

In practice, work processes and protocols provide sufficient flexibility to adapt to different situations.

*Protocols exist to be deviated from. They serve as guidelines for practice and are adjusted based on the needs of the patient. The patient always takes precedence, indeed.*

(P11)

Resilience in maintaining safety involves considering the patient as a whole and their social system, delivering tailored care, and making necessary adjustments to ensure safety. A supportive, positive, and stable team environment with open communication is considered to be crucial for establishing a solid foundation in their work.

### 3.5. Education and Competence Testing

Nurses believe that new colleagues should be required to undergo training to standardise the quality of care and periodic and that mandatory testing should be required for all members of technical homecare teams.

*That’s good, that’s necessary. If you want to stay up to date with all developments, you need to keep assessing yourself, because otherwise you can become unconsciously incompetent, and that is the most dangerous thing there is.*

(P11)

They emphasise the importance of testing by an external organisation due to the high level of complexity involved, considering it more valuable than peer-to-peer assessments.

Testing all skills is seen as challenging due to the large number involved. Teams organise this in various ways, such as group assessments, peer learning and exchanging feedback, preparing for the most critical tasks while testing a random selection, or holding sessions where skills are tested within a few hours.

*If you claim that frequently performed procedures don’t need to be tested, those are precisely the ones for which you no longer consult the protocol–meaning you may miss updates or changes. I believe it is exactly those skills you perform most often that should be tested.*

(P4)

Periodic retraining is generally mandatory, for example, every three years, including testing and sometimes clinical reasoning. Clinical reasoning requires explaining the background or potential complications. The lack of assessment of clinical reasoning in some teams is seen as a risk factor.

*It is important, as long as you know what you’re doing, and especially to know what to do if things go wrong. Often, the technique itself can be learned, but you need to understand the reasoning behind it, and I think you’re very much responsible yourself for knowing whether you’re competent and authorised to do it.*

(P2)

Practical skills are taught through on-the-job learning, e-learning modules, and mandatory skills lab training. Nurses are actively trained when new developments arise or protocols change.

Nurses state that competence and qualification can be ensured through an adequate system of training and assessment, with regular updates to ensure patient safety. The introduction of the ‘Technical homecare nurse training programme (TTV course)’ [35] can significantly contribute to professional development, according to nurses. Ideally, the ‘Technical homecare nurse training programme (TTV course)’ should be nationally endorsed for all technical homecare team members. Some teams or employers already require this training.

*Because that [programme] was developed somewhat in response to a gap—well, there was room for improvement in technical homecare—and this course was created in collaboration with the professional association Nurses & Carers Netherlands (V&VN).*

(P3)

Nurses see the responsibility of remaining qualified and competent as professionals as partly their own and partly that of their employer. They believe they should critically assess their ability to perform a task and seek training when necessary. Key conditions for maintaining competence include management support for training opportunities, time to attend courses, membership of a professional technical nurses’ association, access to national conferences, and allocated funding for professional development.

*I have always worked in a hospital, where there were many opportunities to attend courses, and plenty of funding and time for training. I’ve noticed that in homecare, these opportunities are significantly more limited.*

(P1)

Additionally, nurses would like to see national standardisation of certain procedures. The national Vilans protocols are considered subordinate to the various more detailed hospital-specific protocols.

In conclusion, key findings/statements are derived from the results (see Table 1).

## 4. Discussion

Based on data from two group interviews, verified by the respondents, this study provides statements and recommendations regarding responsibilities at various levels related to quality and safety management, as well as educational needs concerning the use of AMTs in homecare. These statements and recommendations, identified within a specific and relatively small group, represent an initial step for further research among a larger and more diverse group. They may also be used for further development at the national level and, potentially, internationally by management and policymakers.

Dutch legislation imposes obligations for safe reporting and learning from healthcare incidents, while allowing organisations the flexibility to design systems tailored to their specific situation [8]. Participating nurses regard an open and safe organisational culture as essential for incident reporting at the organisational level. The law [8] requires incidents to be discussed with the patient and documented in the patient’s file. However, participating nurses feel this conflicts with fostering a safe reporting culture. They are more comfortable reporting incidents within their team than via the organisation’s formal protocol. As a consequence, the likelihood of reporting would be increased. In addition to a formal reporting system, discussing incidents at a team level is also seen as highly important. These findings suggest that such team-based discussions should be formally acknowledged and supported within organisational processes.

Teams must be adequately supported in order for them to report incidents in line with organisational protocols, as shown in our study. This includes allocating sufficient time, improving digital forms, and providing more regular feedback on outcomes, so that nurses understand how their reports are being used. This aligns with earlier studies [36,37,38]. Participating nurses are more accepting of reporting systems that demonstrate a consistent approach and visibly lead to safer practices. At the national level, it is advised to develop guidelines that are more uniform across homecare organisations and that are in alignment with hospitals regarding the deployment of technology in homecare. This ensures clear responsibilities, enhances patient safety, and promotes a culture of continuous quality improvement [39].

Participating nurses indicated that distinguishing between high-risk and low-risk incident reports can improve the efficiency and effectiveness of safety management concerning AMTs, facilitating better prioritisation of resources, targeted preventive measures, and more effective analysis of patterns [40]. This approach explicitly assigns responsibility to teams for managing low-risk incidents and implementing appropriate improvements. Based on the results, it is recommended that this be included in the (re)design of safety management systems, with support provided to teams to explore incidents in depth for learning purposes.

To enable effective reporting and discussion of incidents, teams must also cultivate a culture of safety and open communication. Participating nurses view a supportive, positive, and stable team environment as essential for building a strong foundation in their work, and they see themselves as active contributors to this. Incident discussion at the team level should be a standing agenda item, as evidenced by the results. Our results also show that each team may require a tailored approach regarding which incidents are addressed and how. However, a previous study within the same organisations revealed that only 16% of AMTs-related incidents were reported in accordance with protocol [5]. Team members can be encouraged to continue the formal reporting of their own mistakes, which is also preferred over reporting those of colleagues.

While problems with AMTs are more commonly discussed than successes, participating nurses clearly express a desire to share positive experiences as well. Some nurse expressed that it would be beneficial to devote more time to explicitly discussing positive examples in care. Another participant noted that this would also be desirable within her organisation, with an addition from another participant highlighting that it provides material to move forward. A further participant mentioned that successes are always acknowledged in her team. This aligns with the Safety II approach to learning from situations that occur correctly. Safety II also provides the opportunity to act proactively on challenges in care processes and to continue delivering safely through adaptations in response to variations in daily practice [41]. This aligns with the view of participating nurses that adaptability is a core skill necessary for working in a technical homecare team and managing the constant variations in their work, making the necessary adjustments to ensure safety. However, by focusing too much on Safety II, the structural causes of incidents may be insufficiently recognised [42]. Homecare organisations should therefore find a balance between learning from incidents (Safety I) and learning from successes and resilience (Safety II).

Dutch legislation requires nurses to be licensed and competent but leaves it to organisations to determine how responsibilities, training, and assessments are implemented [43]. We recommend that teams be actively involved in designing effective and efficient education and testing systems. Organisations should support this by helping teams maintain their competence. Participating nurses specifically request external assessments, management support, funding, and sufficient time for training. A previous study [6] found that many homecare nurses working with AMTs believed their education provided insufficient focus on patient safety, risks, incidents, and the specific context of homecare. This underscores the need for targeted training programmes in these areas. Mandatory external assessments and targeted training enhance the quality and safety of technical homecare but entail substantial costs and organisational challenges. Mandatory assessment and training require time, which temporarily reduces the availability of nurses; however, the continuity of care in practice must be maintained throughout [44]. In addition, effective regional collaboration with educational institutions and AMT suppliers is important, with clear agreements regarding responsibility for assessment, monitoring, and quality assurance [45,46]. Moreover, competency requirements, recertification, and professional development should be structurally embedded in HR policy. A cultural shift within the organisation is needed towards one that favours continuous learning, accountability, and reflection, rather than one-off competence assessments [47].

Participating nurses also see it as their own responsibility to maintain competence in using AMTs. They can assess their own learning needs and take appropriate steps to remain up to date. However, earlier research showed that some nurses use AMTs despite feeling insufficiently competent, posing a clear safety risk [6]. Moreover, a significant number of nurses only participate in occasional or voluntary retraining (and some not at all)—another risk factor. Introducing a system of regular, mandatory skills assessments for all members of technical homecare teams could help mitigate these risks. Participating nurses recommend compulsory participation in the ‘Technical homecare nurse training programme’, with this compulsory participation ideally stated in national guidelines. At the same time, there is a growing focus on self-directed learning and competence management. This shift highlights the need to strike a new balance between mandatory training—which can be a burden on administration—and encouraging self-management of ongoing professional development.

A major strength of this study is that participants in the group interviews came from the same organisations involved in earlier research on this subject. These previous findings served as the basis for this study, resulting in a more comprehensive set of conclusions. Another strength is that all geographical and economic regions across the country are represented among the participants, encompassing both urban and rural areas. In addition, with a view to the future development of greater specialisation among nurses working with AMTs in homecare, this study brought together respondents with extensive specialised experience in this field. These respondents reached consensus on a number of statements for which no consensus previously existed at either national or international level and which can serve as a first step towards further and broader development.

A limitation of this study concerns the sample size, as it included 11 participants. However, the preliminary statements were then presented to all 25 nurses who had expressed an interest in participating in a group interview, in order to reach consensus. Furthermore, recruitment was by invitation. The call for participation may have predominantly attracted nurses with strong opinions or critical views, which could have resulted in self-selection bias. Additionally, no male nurses were able to participate on the specified dates and times. The group interviews could, at the specified time, only be attended by nurses from technical homecare teams, with no generalist homecare nurses. A suggestion for further research is a follow-up study with a larger and more representative group of participants. The reasons for participant dropout were not systematically recorded, preventing analysis of potential dropout bias. These factors limit the generalisability. Finally, there are differences between online and face-to-face interactions, which may lead to measurably different outcomes [48,49]; holding the group interviews online may have created some psychological distance between participants and hindered the exploration of sensitive issues. However, the online format was the most practical given the geographical spread of participants.

## 5. Conclusions

This study serves as the final step in examining quality and patient safety practices concerning AMTs in homecare. The findings confirm previous results indicating strong awareness among homecare nurses regarding quality and patient safety. It is now essential to develop incident reporting systems in which homecare nurses feel safe and supported. Team-level systems should be perceived as effective and efficient. Interconnected learning cycles at organisational, team, and individual levels can strengthen collaboration across these layers. In doing so, homecare organisations can progress towards becoming learning organisations—where quality and safety are approached more proactively than reactively.

The consensus statements contain a number that are relevant for healthcare managers and policymakers, particularly those relating to the allocation of tasks and responsibility at various levels, organisational, team and individual, in relation to safety management, incident reporting and analysis, and improvement actions. Practical implications are indicated in the set of statements. It is extremely important to respect the practice of homecare nurses to discuss and resolve safety issues at the team level. Reaching further consensus on a national safety management guideline for AMT use in homecare, as well as on related educational needs, will certainly contribute to safer technology use in this setting.

## Figures and Tables

**Table 1:** Key findings/statements per theme following the results.

Safety Management System Regarding Advanced Medical Technologies (AMTs)	
1.	Essential components of a safety management system concerning AMTs in homecare are (1) an adequate incident reporting system and (2) appropriate training and assessment, according to nurses.
2.	For nurses in homecare, (Vilans) protocols, framework agreements with other care institutions (hospitals), and the professional code provide the most direction for safe practices regarding AMTs. The safety management system integrates these elements into organisational policy.
**Levels and processes regarding incident reporting**	
3.	Distinguishing between high-risk and low-risk incident reports is considered to enhance the efficiency and effectiveness of safety management for AMTs in homecare.
4.	Nurses feel more comfortable reporting incidents at the team level than through the organisation’s formal protocol.
5.	Discussing incidents within the team, rather than submitting a formal report according to the organisation’s protocol, increases the likelihood of incidents being reported, according to nurses.
6.	Discussing incidents within the team is considered highly important but requires additional time to facilitate deeper analysis of situations and promote learning as a team.
7.	The fact that patients and their families can access the client record and see that an incident has occurred is experienced as a barrier by nurses to making a formal report; this creates tension within the development of a safe reporting culture.
**Safety II in technical homecare teams**	
8.	What goes wrong with AMTs is more likely to be discussed within the team than what goes well.
9.	When positive examples are addressed, this tends to be more implicit than explicit, and they are not consciously reflected upon as a team. Nurses do, however, consider it desirable to systematically discuss positive examples.
10.	A core skill of working in a technical homecare team is considered to be the ability to continuously adapt to the constant variations in the work.
11.	Resilience in working as safely as possible involves viewing the individual in their entirety, including their social system, providing tailored solutions, and making adjustments to ensure certain safety standards are met, in nurses’ views.
**Competence and authority of homecare nurses**	
12.	Nurses believe that assessment/testing for technical homecare teams should be periodic and mandatory for everyone.
13.	Nurses believe it is important that the assessment of skills related to AMTs in homecare is carried out by an external organisation, due to the high complexity. External assessment clearly provides added value over peer-to-peer assessment.
14.	Nurses believe that, particularly for new staff, further mandatory training should be implemented to standardise the quality of practice.
15.	The fact that not all teams are assessed on clinical reasoning is seen as a risk factor for patient safety by nurses.
16.	Nurses believe that it is partly their own responsibility as professionals to remain competent and skilled and partly the responsibility of the organisation.
17.	Nurses consider that they should primarily assess for themselves what they need to stay up to date and take action accordingly.
18.	All nurses in a technical homecare team must undergo the ‘Technical homecare nurse training programme (TTV course)’, according to nurses; this will contribute to their expertise and competence and consequently to safety.
19.	Nurses express the need for more consistent national guidelines regarding certain procedures between homecare organisations and hospitals, since, in practice, the national Vilans protocols are subordinate to various specific hospital protocols.

## Data Availability

The dataset supporting the conclusions of this article is included within the article. Complete raw data, the transcribed group interviews, generated and analysed during the current study are available, although in Dutch, upon reasonable request from the corresponding author.

## Supplementary Materials

The following supporting information can be downloaded at: https://www.mdpi.com/article/10.3390/healthcare14040529/s1, Supplementary File SA: Semi-structured discussion guideline group interviews. Supplementary File SB: Participant characteristics group interviews.

## Abbreviations

The following abbreviations are used in this manuscript:

AMTs	Advanced Medical Technologies
