# Supplementary material for: Quality and Safety Management of Advanced Medical Technologies in Homecare in The Netherlands: A Qualitative Study on Consensus Development Regarding Approaches and Continuing Professional Education
Source: Healthcare (Basel). 2026 Feb 20;14(4):529. doi: 10.3390/healthcare14040529 (PMC12940245; doi:10.3390/healthcare14040529)
Supplement: Supplementary file 1 [file healthcare-14-00529-s001.zip › Supplementary File SB.pdf]

Supplementary File SB. Participant characteristics group interviews.

Table SA.1.
Participant characteristics group interviews.

Participant	Group Interview	Gender	Nature of the homecare team	Possible side activities alongside their job
P1	1	F	Specialist technical team	Unknown
P2	1	F	Specialist technical team	- Also provides specialist care in nursing homes, such as palliative sedation and PICC line care.
P3	1	F	Specialist technical team	- Serves on the board of the Technical Home Care Department.<br>- Nurse at the Professional Association of Nurses & Caregivers Netherlands (V&VN).<br>- Works at Carend, a platform dedicated to palliative care.
P4	1	F	Specialist technical team	- Contributes to the development of nursing education.<br>- Contributes to the first Dutch guideline on central venous catheters.<br>- Conducting assessments.<br>- Provides training sessions/education.
P5	1	F	Specialist technical team	- Works on projects such as hospital-at-home care and care at the beginning of district nursing, to encourage more organisations to collaborate.
P6	1	F	Specialist technical team	Unknown
P7	1	F	Specialist technical team	- Instructor of nursing technical procedures within the organisation.
P8	2	F	Specialist technical team	Unknown
P9	2	F	Specialist technical team	- Coordinator of the team, but also still works alongside the team.
P10	2	F	Specialist technical team	- Works as a senior nurse and spends several days a week on the rounds herself.
P11	2	F	Specialist technical team	Unknown

P = participant
F = female
