# Supplementary material for: Quality and Safety Management of Advanced Medical Technologies in Homecare in The Netherlands: A Qualitative Study on Consensus Development Regarding Approaches and Continuing Professional Education
Source: Healthcare (Basel). 2026 Feb 20;14(4):529. doi: 10.3390/healthcare14040529 (PMC12940245; doi:10.3390/healthcare14040529)
Supplement: Supplementary file 1 [file healthcare-14-00529-s001.zip › Supplementary File SA.pdf]

Supplementary File SA.Semi-structured discussion guideline group interviews

Introduction:
Many organisations have a safety management system in place for (advanced) medical technology. The aim of this system is to embed patient safety into practice. It consists of a set of concrete activities and procedures related to the introduction, use, and decommissioning of medical technology. Aspects associated with this include PDCA, maintenance, user authorization and competence, training, and learning from incidents during safety rounds.

1. What do you consider to be the core processes of a safety management system for (advanced) medical technology? Please elaborate.

2. Is an organisation-wide technology management system necessary/essential for the safe use of advanced medical technology? Why?

Introduction
Previous research among home care nurses shows that only 16% of incidents are formally reported according to the organisation's protocol. However, in over 70% of cases, incidents are discussed within the team (1).

3. Which incident reporting processes, in your opinion, could be better structured at the organisational level, and which would be preferable at the team level?
   a. Why?
   b. What conditions are necessary for this:
      - At the organisational level
      - At the team level
      - At the individual level

4. How, in your opinion, should this process be structured at the team level, and how could it be optimised?

Introduction:
So far, the focus has primarily been on Safety I: Safety I involves investigating things that go wrong by reporting and analysing all (near-)incidents and adverse events. Work in practice is generally documented in processes and protocols.
However, there is a trend to approach safety from the perspective of Safety II: looking at what goes well in daily practice on the work floor.

5. Are positive examples of what goes well in the use of complex medical technology also discussed or shared among colleagues?
   a. Do you build on positive examples? How?
   b. Are there reflection moments with the team?

6. How do you ensure that Safety II is implemented, and what is needed for this?
   a. How do you and your teams manage to continuously adapt to the constant variations in your work?
   b. And what constitutes your resilience to work as safely as possible?

Introduction

Previous studies (2,3) in this research show that regarding advanced medical technology:

- Practical skills are not always taught,
- Continuing education is not always mandatory,
- Not all skills are assessed.

7. To what extent do you believe it is necessary for all skills to be assessed, continuing education to be mandatory, and practical skills to be taught?
   (Not as an administrative obligation, but as personal responsibility – to what extent is this feasible?)

8. How do you ensure that the competence and authorisation of nurses regarding advanced medical technology are met?
   a. What conditions are necessary within your team to address these factors?
   b. What is needed at the organisational level?
   c. What might this imply at a national level?

Finally, are there any aspects related to the safe use of advanced medical technology in homecare that have not been addressed, and that you would like to mention?

References

1. ten Haken I, Ben Allouch S, van Harten WH. Reporting incidents involving the use of advanced medical technologies by nurses in home care: a cross-sectional survey and an analysis of registration data. BMJ Qual Saf. 2020 May;30(5):380–7.

2. ten Haken I, Ben Allouch S, van Harten WH. Education and training of nurses in the use of advanced medical technologies in home care related to patient safety: A cross-sectional survey. Nurse Education Today. 2021 May;100:104813.

3. ten Haken I, Ben Allouch S, van Harten WH. Quality and safety management of advanced medical technologies in home care organizations in the Netherlands: A qualitative survey at the tactical level. BMC Nursing (under submission). 2025;
